# Immune-Related LncRNAs Affect the Prognosis of Osteosarcoma, Which Are Related to the Tumor Immune Microenvironment

**DOI:** 10.3389/fcell.2021.731311

**Published:** 2021-10-07

**Authors:** Qingshan Huang, Yilin Lin, Chenglong Chen, Jingbing Lou, Tingting Ren, Yi Huang, Hongliang Zhang, Yiyang Yu, Yu Guo, Wei Wang, Boyang Wang, Jianfang Niu, Jiuhui Xu, Lei Guo, Wei Guo

**Affiliations:** ^1^Musculoskeletal Tumor Center, Peking University People’s Hospital, Beijing, China; ^2^Beijing Key Laboratory of Musculoskeletal Tumor, Peking University People’s Hospital, Beijing, China; ^3^Laboratory of Surgical Oncology, Peking University People’s Hospital, Beijing, China

**Keywords:** osteosarcoma, lncRNA, tumor immune microenvironment, immune escape, metastasis

## Abstract

**Background:** Abnormal expression of lncRNA is closely related to the occurrence and metastasis of osteosarcoma. The tumor immune microenvironment (TIM) is considered to be an important factor affecting the prognosis and treatment of osteosarcoma. This study aims to explore the effect of immune-related lncRNAs (IRLs) on the prognosis of osteosarcoma and its relationship with the TIM.

**Methods:** Ninety-five osteosarcoma samples from the TARGET database were included. Iterative LASSO regression and multivariate Cox regression analysis were used to screen the IRLs signature with the optimal AUC. The predict function was used to calculate the risk score and divide osteosarcoma into a high-risk group and low-risk group based on the optimal cut-off value of the risk score. The lncRNAs in IRLs signature that affect metastasis were screened for *in vitro* validation. Single sample gene set enrichment analysis (ssGSEA) and ESTIMATE algorithms were used to evaluate the role of TIM in the influence of IRLs on osteosarcoma prognosis.

**Results:** Ten IRLs constituted the IRLs signature, with an AUC of 0.96. The recurrence and metastasis rates of osteosarcoma in the high-risk group were higher than those in the low-risk group. *In vitro* experiments showed that knockdown of lncRNA (AC006033.2) could increase the proliferation, migration, and invasion of osteosarcoma. ssGSEA and ESTIMATE results showed that the immune cell content and immune score in the low-risk group were generally higher than those in the high-risk group. In addition, the expression levels of immune escape-related genes were higher in the high-risk group.

**Conclusion:** The IRLs signature is a reliable biomarker for the prognosis of osteosarcoma, and they alter the prognosis of osteosarcoma. In addition, IRLs signature and patient prognosis may be related to TIM in osteosarcoma. The higher the content of immune cells in the TIM of osteosarcoma, the lower the risk score of patients and the better the prognosis. The higher the expression of immune escape-related genes, the lower the risk score of patients and the better the prognosis.

## Introduction

Osteosarcoma is the most common primary malignant bone tumor, most commonly occurring in adolescents and children (Huang et al., 2021). Its incidence is about 4.4 per million, accounting for 5% of all childhood malignancies (Han et al., 2021). Nearly 60% of osteosarcomas occur in the femur, tibia, and pelvis (Zhou and Mu, 2021). Osteosarcoma has the characteristics of high malignancy, rapid growth, and easy metastasis. Pulmonary metastasis is one of the major factors leading to the poor prognosis of osteosarcoma, and more than 20% of patients with osteosarcoma have had pulmonary metastasis at the time of diagnosis (Anderson, 2016). Therefore, it is important to clarify the causes and mechanisms affecting the prognosis of osteosarcoma to prolong the survival time of osteosarcoma.

LncRNA regulates gene transcription, translation, editing, and other biological processes (Qian et al., 2019). Its abnormal expression is closely related to the occurrence and metastasis of tumors (Kornfeld and Bruning, 2014; Bartonicek et al., 2016). It has been confirmed that multiple lncRNAs can promote the occurrence and metastasis of osteosarcoma through competitive inhibition of miRNA expression (Pan et al., 2021; Zhou and Mu, 2021). Notably, lncRNAs can regulate the development and activation of a variety of immune cells (Atianand et al., 2017). The tumor microenvironment is composed of extracellular matrix, mesenchymal cells, immune cells, and other components, and plays an important role in the occurrence of tumors (Pitt et al., 2016; De Nola et al., 2019). The immune cells in the tumor microenvironment are closely related to the treatment and prognosis of tumors. Studies have shown that lncRNA can promote tumor-associated macrophage polarization to regulate the proliferation and migration of tumor cells (Zhao et al., 2021). LncRNA NKILA can also act on T cells to promote the immune escape of tumor cells (Huang et al., 2018). LncRNA THRIL has also been shown to regulate TNF-α expression and participate in immune response in osteosarcoma (Xu et al., 2020). Therefore, these lncRNAs mediated tumor immune microenvironment (TIM) regulation may play an important role in the metastasis and prognosis of osteosarcoma.

In this study, immune-related lncRNAs (IRLs) signature was constructed from the publicly available RNA-Seq dataset to evaluate the prognosis of osteosarcoma. In particular, the role of IRLs signature in osteosarcoma metastasis was evaluated and validated *in vitro*. Because the metastasis of osteosarcoma is an important factor affecting its poor prognosis. In addition, this study further explored the role of TIM in the influence of IRLs on the progression of osteosarcoma.

## Materials and Methods

### Data Collection and Processing

Osteosarcoma expression spectrum and clinical information from the TARGET database.^1^ Samples were selected and data were processed by the following steps: (1) samples with both expression profiles and prognostic information were selected; (2) delete the samples with a survival time of 0 months; (3) according to the human gene annotation file (version GRCH38.p13), the ID of the gene of the osteosarcoma samples was converted into the gene symbol; and (4) there were multiple expression levels of the same gene in the expression profile, and the mean expression level was taken.

### Co-expression Analyses of Immune-Related LncRNAs

A gene set named “IMMUNE RESPONSE TO TUMOR CELL” was download from Molecular Signatures Database.^2^

Correlation analysis was conducted between all lncRNAs and the gene set, and IRLs with a correlation coefficient greater than 0.6 were obtained.

### Construction Immune-Related LncRNAs Prognostic Signature

LncRNAs with expression variance greater than 0.2 in IRLs were screened. Univariate Cox regression analysis was performed on these lncRNAs and those with *P*-value less than 0.5 were screened (Sveen et al., 2012). Iterative LASSO regression was used to identify high-frequency lncRNA (Zhang et al., 2021). Through 1000 iterations, a total of 11 lncRNAs with a frequency greater than 300 were screened out. These lncRNAs were incorporated into the Cox regression analysis one by one until the AUC value of IRLs signature reached the maximum. The risk score of each osteosarcoma sample was calculated using the predict function. The patients were divided into a high-risk group and low-risk group using the optimal risk score cut-off value and the Survminer R package was used for survival analysis.

### Single Sample Gene Set Enrichment Analysis and Gene Set Enrichment Analysis

Fifty immune cell gene sets (Bindea et al., 2013; Cheng et al., 2013; Newman et al., 2015; Senbabaoglu et al., 2016), stromal cell gene set, and total immune cell gene set included in this study (Yoshihara et al., 2013) came from previous studies. Single sample gene set enrichment analysis (ssGSEA) of these gene sets were performed using Gene Set Variation Analysis (GSVA) R package (Wang et al., 2021), and the results of ssGSEA were normalized. KEGG pathway enrichment and GO function enrichment analysis were performed using GSEA software (version 4.0.1). Gene sets of KEGG and GO (C2.Cp.KEGG.v7.1 and C5.All.V7.1.Symbols) download from Molecular Signatures Database (see text footnote 2).

### Construction of the Nomogram

The risk score and osteosarcoma features such as age, gender, recurrence, metastasis, and tumor site were used to construct a nomogram (Iasonos et al., 2008; Wang et al., 2013) to intuitively evaluate the prognosis of patients with osteosarcoma. The decision curve was used to verify the accuracy of the nomogram.

### Estimation of Tumor Microenvironment Score

ESTIMATE algorithm was used to evaluate stromal score, immune score, and tumor cell purity of osteosarcoma (Hu et al., 2021). Kruskal–Wallis was used to analyze the differences in the stromal score, immune score, Estimate score, and tumor cell purity of different risk score osteosarcomas.

### Cell Culture and Transfection

Human osteosarcoma cell lines KHOS and 143B were derived from American Type Culture Collection (ATCC, VA, United States). 143B cells were cultured in DMEM medium (HyClone, UT, United States) containing 10% fetal bovine serum (FBS, Gibco, NY, United States), and KHOS cells were cultured in RPMI-1640 medium (HyClone, UT, United States) containing 10% FBS. Si-AC006033.2 was obtained from Gemma Gene (Suzhou, China) (sequences: 5′-GCAGCUGCUUUGACAGUUUTT-3′). Lipofectamine 3000 (Invitrogen, CA, United States) was used for transfection. The transfection process was carried out according to the instructions.

### Reverse Transcription-Quantitative Polymerase Chain Reaction

RNA extraction from osteosarcoma cell lines was performed using TRIzol (Invitrogen, CA, United States). GAPDH was selected as an endogenous control. The primers of AC006033.2 and GAPDH are shown in Supplementary Table 1.

### Cell Proliferation Assay (Cell Counting Kit-8 and 5-Ethynyl-2′-Deoxyuridine)

Osteosarcoma cells were cultured in 96-well plates with a cell density of 3000 cells per well. A total of 10 μL Cell Counting Kit-8 (CCK-8) solution (Beyotime, Shanghai, China) was added at 0, 24, 48, and 72 h, respectively. OD values were measured at 450 nm wavelength.

Osteosarcoma cells were inoculated in six-well plates and cultured for 12 h, 5-ethynyl-2′-deoxyuridine (EdU, Beyotime, Shanghai, China), was added. The final concentration of EdU was 10 μM. It was incubated in an incubator at 37°C for 2 h and fixed with 4% paraformaldehyde. A total of 1 mL of osmotic solution was added to each well and incubate at room temperature for 15 min. A total of 0.5 mL click reaction solution was added to each well and incubate at room temperature in dark for 30 min. Finally, the nuclei were stained with DAPI (Beyotime, Shanghai, China).

### Wound Healing Assay and Transwell Invasion Assay

The wound-healing assay was performed in a six-well plate. Scratches were made with the tip of a sterile pipette. The changes of scratches at 24 h were observed under a microscope. Image-Pro Plus 6.0 software (Media Cybernetics, United States) was used to calculate the area change of the scratches. Invasion experiments were performed in transwell chambers (Corning, NY, United States) with an 8 μm pore diameter membrane. The upper layer of the chamber was added with matrigel (BD, NJ, United States). The number of osteosarcoma cells inoculated was 1 × 10^5^ pre well. The upper chamber was cultured with serum-free medium, and the lower chamber was cultured with a 700 ‘L complete medium. After 48 h, the cells in the upper part of the basement membrane were erased, and the cells in the lower part of the basement membrane were fixed and stained.

### Statistical Analysis

The statistical software R (version 3.6.1) was used for data analysis and image production. The Chi-square test was used to compare differences in recurrence rates or metastasis rates of osteosarcoma. Spearman correlation analysis was used to evaluate the correlation between the expression level of lncRNAs, the correlation between risk score and lncRNA expression level, or the correlation between lncRNA expression level and immune cell content. The Kruskal–Wallis test was used to analyze the differences in immune cell content, tumor microenvironment score, or gene expression level. Kaplan–Meier survival analysis was used to evaluate prognostic differences between different risk scores or between different lncRNAs expression levels in osteosarcoma. Univariate Cox regression analysis and multivariate Cox regression analysis were used to evaluating the effects of lncRNA, clinical characteristics, or risk score on the prognosis of osteosarcoma. Two-tailed *P*-values were used, and the statistical significance was set at *P* < 0.05.

## Results

### Data Processing and Co-expression Analyses of Immune-Related LncRNAs

Sequencing data and clinical data were downloaded from the TARGET database and expression levels of all samples were combined into an expression profile. The names of samples with both clinical and sequencing data are shown in Supplementary Table 2. Follow the above method, 95 osteosarcoma samples were included in the study. The expression levels of all lncRNA were shown in Supplementary Table 3. Correlation analysis was conducted between all lncRNAs and the gene set (IMMUNE RESPONSE TO TUMOR CELL), and 4986 IRLs were obtained (Supplementary Table 4).

### Construction of Immune-Related LncRNAs Prognostic Signature

**FIGURE 1:**
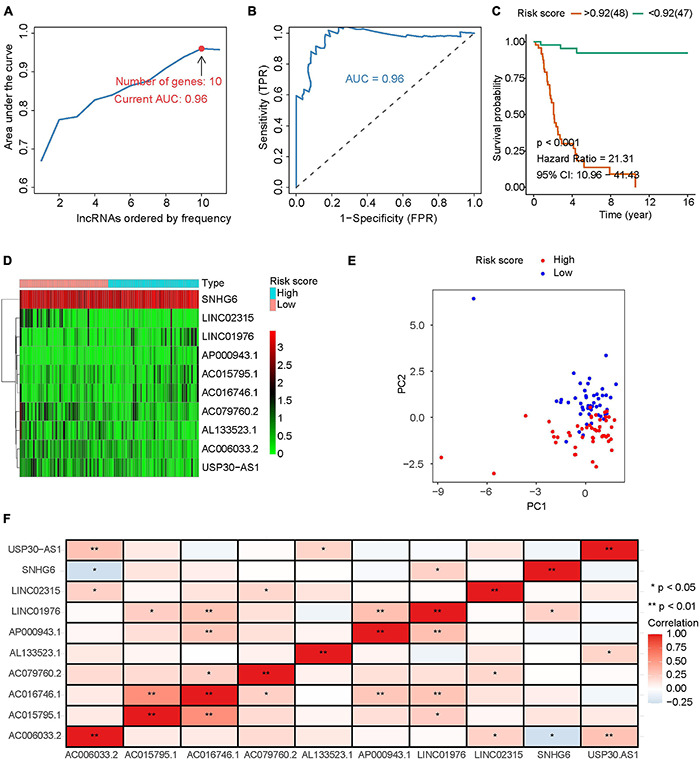
Construction of IRLs prognostic signature. **(A)** Iterative LASSO and Cox regression analysis are used to screen IRLs signature with the best AUC. **(B)** The ROC curve with the best AUC. **(C)** Osteosarcomas were grouped according to the optimal cut-off value of risk score, and survival analysis was performed. **(D)** Expression levels of 10 lncRNA in IRLs signature. **(E)** PCA analysis was performed with IRLs signature. **(F)** Correlation analysis among 10 IRLs in IRLs signature.

There were 4986 lncRNAs with variances greater than 0.2 expressed in IRLs. Univariate Cox regression analysis was performed on these IRLs, among which 1743 IRLs with *P*-value less than 0.5 were identified. Iterative LASSO regression was used to identify high-frequency lncRNAs. One thousand iterations were executed, and 11 lncRNAs with a frequency greater than 300 were screened out. These lncRNAs were incorporated into the Cox regression analysis one by one until the AUC of ROC reached the maximum. At this point, the number of lncRNAs was 10 and the AUC was 0.96 (Figures 1A,B). The predict function was used to calculate the risk score of each osteosarcoma sample, and the osteosarcoma patients are divided into the high-risk group (*n* = 48) and low-risk group (*n* = 47) according to the optimal cut-off value (0.92) of risk score. Kaplan–Meier survival analysis showed that the lower risk score was associated with a better prognosis for osteosarcoma (Figure 1C). The distribution of 10 IRLs expression levels with the change of risk score was shown in Figure 1D. The results of principal component analysis (PCA) showed that IRLs signature can achieve better dimension reduction (Figure 1E). Correlation analysis results showed that the expression levels of these lncRNAs were not highly correlated (Figure 1F), which further demonstrated the rationality of IRLs signature.

### The Role of Immune-Related LncRNAs Signature in the Prognosis of Osteosarcoma

**FIGURE 2:**
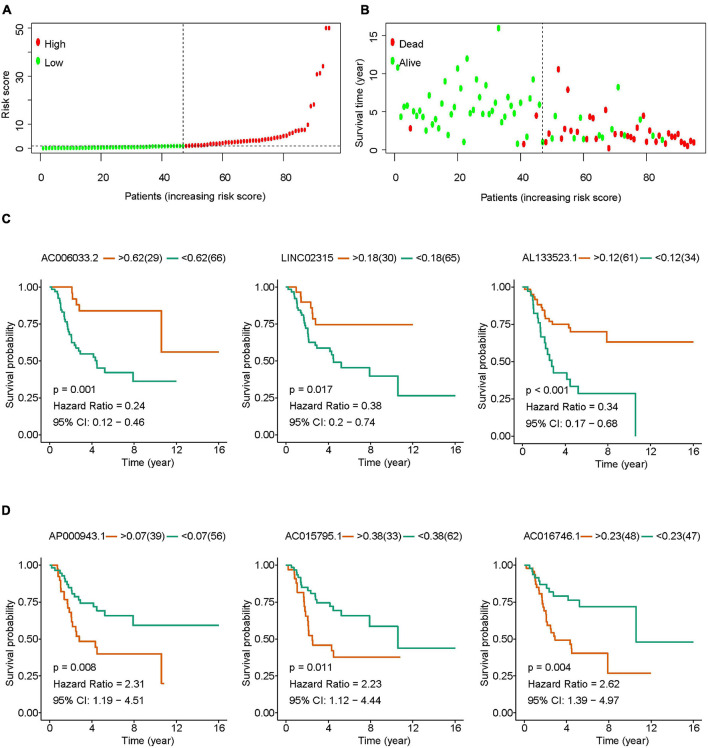
The role of IRLs signature in the prognosis of osteosarcoma. **(A)** Osteosarcoma was divided into high-risk group and low-risk group according to the optimal cut-off value of risk score. **(B)** Relationship between risk score and survival states in patients with osteosarcoma. **(C,D)** Osteosarcomas were grouped according to the optimal cut-off value of lncRNAs expression in IRLs signature, and survival analysis was performed.

Osteosarcoma patients were divided into high-risk group and low-risk group according to the optimal cut-off value of risk score (Figure 2A). With the increase of risk score, the mortality rate of osteosarcoma increased significantly (Figure 2B). Results of survival analysis showed that osteosarcoma with high expression of AC006033.2, LINC02315, AL133523.1, USP30-AS1, or AC079760.2 had a better prognosis (Figure 2C and Supplementary Figure 1A). High expression of AP000943.1, AC015795.1, AC016746.1, LINC01976, and SNHG6 was not conducive to the prognosis of osteosarcoma (Figure 2D and Supplementary Figure 1B). Cox regression analysis showed that AL133523.1, AC079760.2, and LINC01976 were independent prognostic factors for osteosarcoma (Supplementary Tables 5, 6 and Supplementary Figure 2).

### The Role of Clinical Features in the Prognosis of Osteosarcoma

**FIGURE 3:**
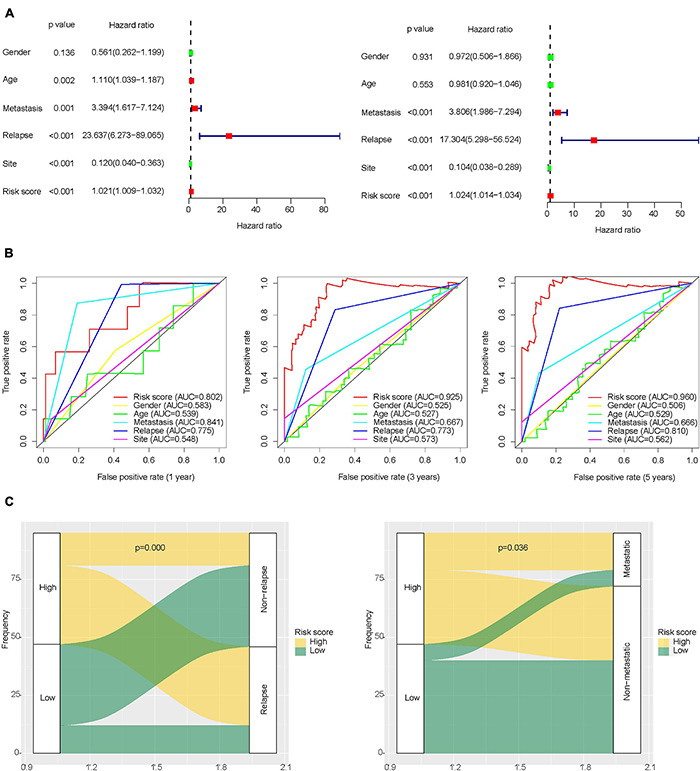
Role of clinical features in the prognosis of osteosarcoma. **(A)** Univariate and multivariate Cox regression analysis of clinical features of osteosarcoma. **(B)** ROC curve for predicting the prognosis of osteosarcoma based on risk score and clinical features of osteosarcoma. **(C)** Differences in metastasis and recurrence rates between the high-risk group and low-risk group.

Univariate and multivariate Cox regression analysis results showed that IRLs signature and the clinical features of osteosarcoma including recurrence, metastasis, and tumor location could all be independent prognostic factors for osteosarcoma (Figure 3A and Supplementary Tables 7, 8). In the prognostic evaluation of osteosarcoma, the predictive performance of IRLs signature was the highest among these features. In the ROC curve, the 1-, 3-, and 5-year AUC values were 0.802, 0.925, and 0.96, respectively (Figure 3B). In addition, the Chi-square test confirmed that the recurrence rates and metastasis rates were higher in the high-risk group than in the low-risk group (Figure 3C). Survival analysis showed a poor prognosis for recurrent, metastatic, and non-limb osteosarcomas (Supplementary Figure 3A), and no significant difference in prognosis between sex and age (Supplementary Figure 3B).

### The Construction and Verification of the Nomogram

**FIGURE 4:**
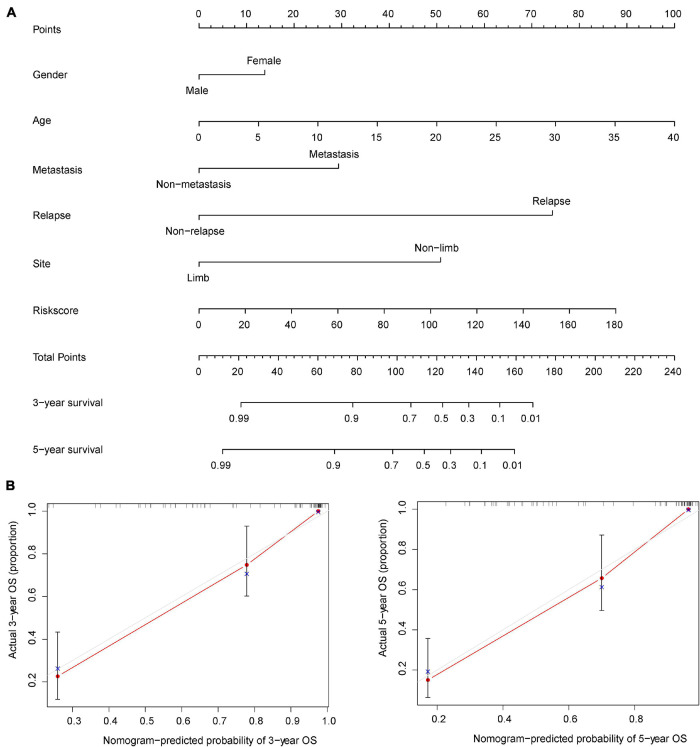
Nomogram for evaluating the prognosis of osteosarcoma at 3 and 5 years. **(A)** The risk score and clinical characteristics of osteosarcoma were used to construct the nomogram. **(B)** Calibration curves were used to verify the accuracy of the nomogram.

The nomogram is widely used to evaluate the prognosis of tumors. It can reduce the statistical prediction model to a probability value. In this study, risk score, gender, age, recurrence, metastasis, and tumor location were integrated to construct a nomogram to evaluate the prognosis of osteosarcoma (Figure 4A). The nomogram showed the predicted survival rates for 3 and 5 years, respectively. Calibration curves showed that the nomogram was able to accurately evaluate the prognosis of osteosarcoma (Figure 4B).

### The Role of Immune-Related LncRNAs Signature in Osteosarcoma Metastasis

**FIGURE 5:**
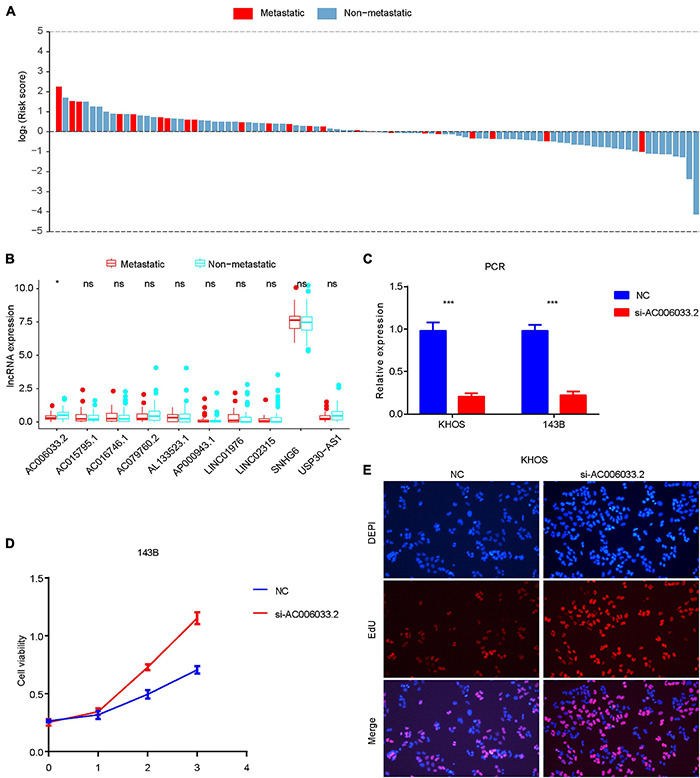
Role of IRLs signature in metastasis and proliferation of osteosarcoma. **(A)** The probability of metastasis of osteosarcoma gradually decreases with the decrease of risk score. **(B)** Differential expression of 10 IRLs in metastatic and non-metastatic osteosarcomas. Only the expression level of AC006033.2 was different. **(C)** Knockdown results of AC006033.2 in osteosarcoma cell lines. **(D,E)** Effects of AC006033.2 knockdown on proliferation of osteosarcoma cells. *P < 0.05 and ***P < 0.001. EdU, 5-ethynyl-2′-deoxyuridine.

**FIGURE 6:**
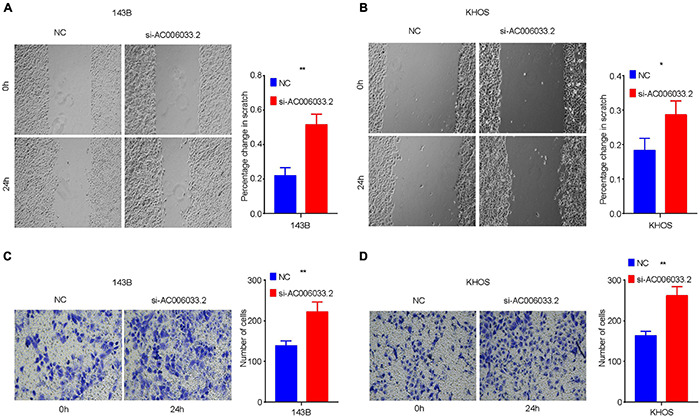
Influence of AC006033.2 on migration and invasion ability of osteosarcoma. **(A,B)** Effects of AC006033.2 knockdown on the migration ability of osteosarcoma cells. **(C,D)** Effects of AC006033.2 knockdown on the invasion ability of osteosarcoma cells. *P < 0.05 and **P < 0.01.

The metastasis of osteosarcoma is one of the most important factors affecting its prognosis. Therefore, this study focused on the role of the IRLs signature in the metastasis of osteosarcoma. The Chi-square test confirmed that the metastatic rate of osteosarcoma was higher in the high-risk group than in the low-risk group (Figure 3C). The waterfall chart also shows that the probability of metastasis gradually decreases with the decrease of risk score (Figure 5A). Therefore, IRLs signature may play an important role in the metastasis of osteosarcoma. The results of the differential analysis showed that only AC006033.2 of the 10 lncRNAs of IRLs signature was different between the metastatic and non-metastatic osteosarcomas, and the expression level of AC006033.2 was low in the metastatic osteosarcomas (Figure 5B). For this reason, AC006033.2 was knocked down and validated in osteosarcoma cell lines. Knockdown efficacy was confirmed by PCR in osteosarcoma cell line 143B and KHOS (Figure 5C). We first conducted a cell proliferation experiment, and the results showed that knocking down AC006033.2 could increase the proliferation ability of osteosarcoma cells (Figures 5D,E). The wound-healing assay results further showed that knocking down AC006033.2 increased the migration ability of osteosarcoma cells (Figures 6A,B). Transwell invasion assay showed increased invasiveness of osteosarcoma cells after AC006033.2 knockdown (Figures 6C,D).

### KEGG Pathway Enrichment and GO Function Enrichment

**FIGURE 7:**
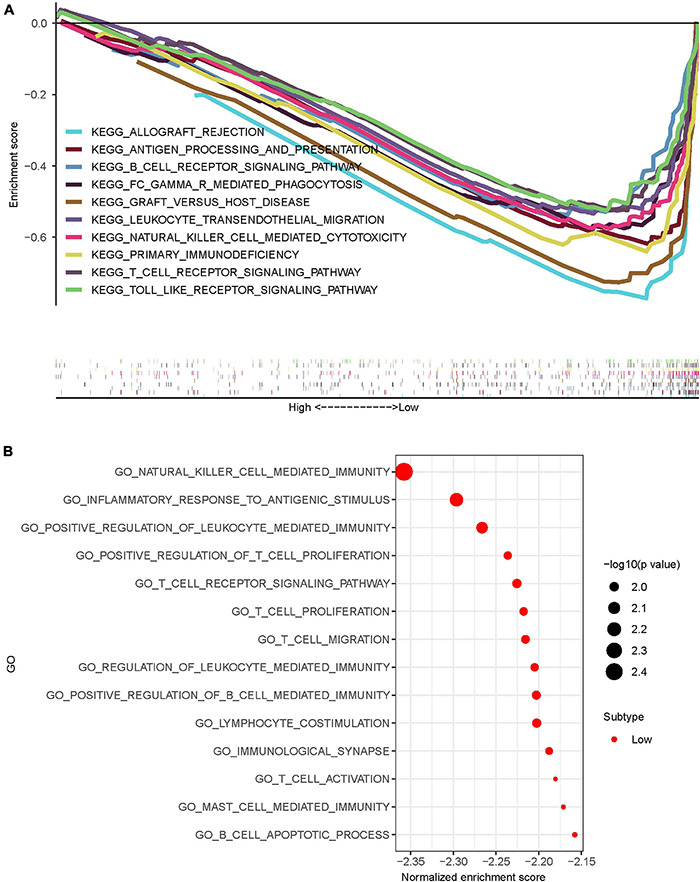
Pathway and functional enrichment analysis of osteosarcoma in the high-risk and low-risk group osteosarcomas. **(A)** KEGG enrichment analysis of osteosarcomas showed that osteosarcomas in the low-risk group were mostly enriched in immune-related pathways. **(B)** GO enrichment analysis of osteosarcomas showed that osteosarcomas in the low-risk group were mostly enriched in immune-related functions.

Gene set enrichment analysis (GSEA) software was used to assess KEGG pathway enrichment in high-risk and low-risk osteosarcomas. The pathways that enriched in low-risk osteosarcomas were mainly immune-related (Figure 7A), while no associated pathways enriched in high-risk osteosarcomas. The better prognosis of osteosarcoma in the low-risk group may be related to the local immune microenvironment.

To further explore the possible role of IRLs signature in osteosarcoma, GO enrichment analysis was performed using GSEA software. The results showed that osteosarcomas in the low-risk group were mainly enriched in immune-related functions (Figure 7B), while the high-risk group osteosarcomas were also not enriched in any meaningfully related functions.

### The Role of Tumor Immune Microenvironment in the Influence of Immune-Related LncRNAs on Osteosarcoma Prognosis

#### Relationship Between Immune-Related LncRNAs Signature and Immune Cell Infiltration

**FIGURE 8:**
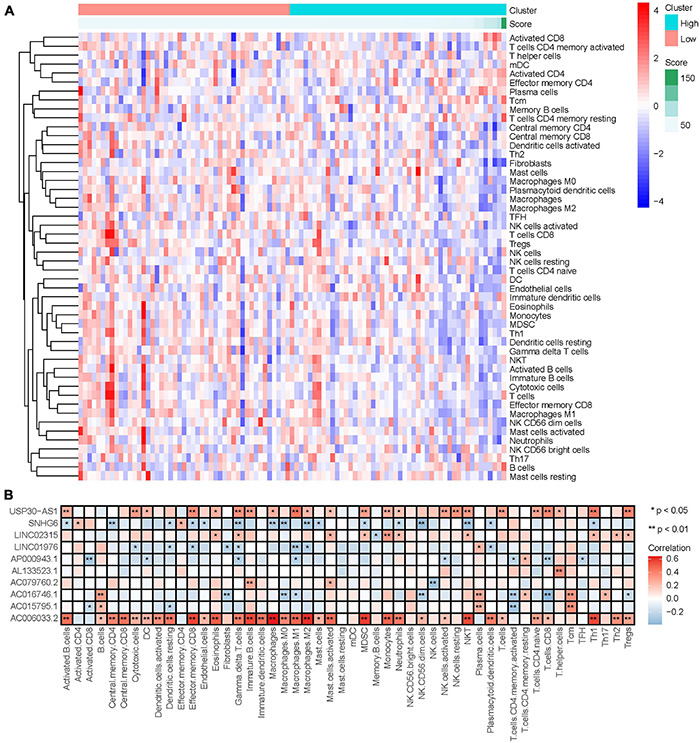
Relationship between IRLs signature and the degree of immune cell infiltration. **(A)** Changes of the degree of immune cell infiltration in osteosarcoma with the change of risk score. **(B)** Correlation analysis between 10 IRLs and the degree of immune cell infiltration.

LncRNA can regulate the development and activation of various immune cells (Atianand et al., 2017). Therefore, our study investigated the relationship between the degree of immune cell infiltration and IRLs signature in the immune microenvironment of osteosarcoma. The results of ssGSEA are shown in Supplementary Table 9, and the content of immune cells in osteosarcoma decreased with the increase of risk score (Figure 8A), and the correlation analysis results showed that risk score was negatively correlated with the content of most immune cells (Supplementary Figure 4), which suggested that the low degree of immune cell infiltration might be an important reason for recurrence and metastasis in the high-risk osteosarcoma. In addition, AC006033.2 was also positively correlated with the content of most immune cells (Figure 8B). Osteosarcoma with low AC006033.2 expression had a low level of immune cell infiltration, which was not conducive to the prognosis of osteosarcoma. This was consistent with the conclusion that AC006033.2 knockdown *in vitro* enhanced the invasiveness of osteosarcoma cells (Figures 6C,D).

#### Relationship Between Immune-Related LncRNAs Signature and Tumor Microenvironment Score

**FIGURE 9:**
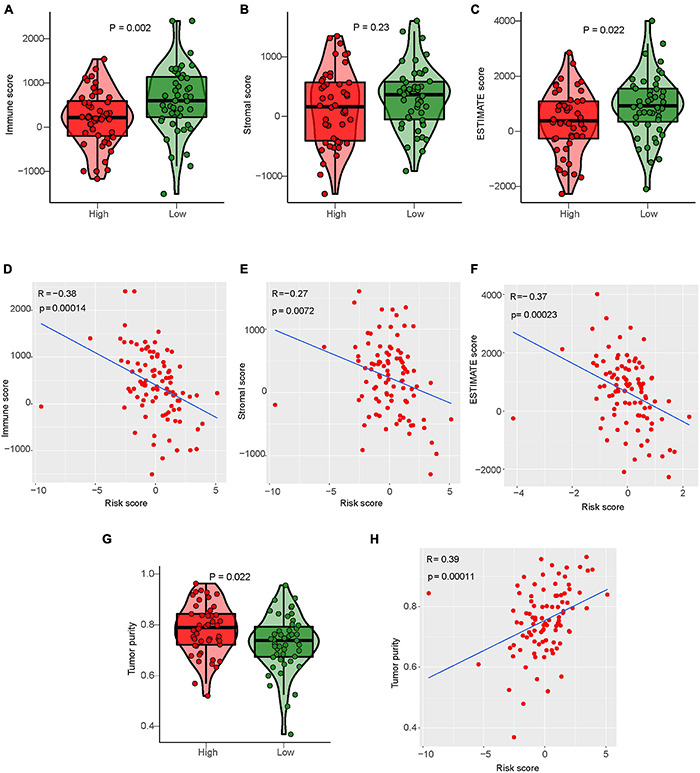
Relationship between IRLs signature and tumor microenvironment score. **(A–C)** Analysis of differences in the immune score, stromal score, and Estimate score between high-risk and low-risk osteosarcoma. **(D–F)** Correlation analysis between risk score and osteosarcoma immune score, matrix score, and Estimate score. **(G)** Analysis of difference in tumor cell purity between high-risk and low-risk osteosarcoma. **(H)** Correlation analysis between risk score and tumor cell purity of osteosarcoma.

ESTIMATE algorithm can be used to evaluate the tumor microenvironment score of osteosarcoma to evaluate tumor stroma content, immune cell content, and tumor cell purity (Supplementary Table 10; Hu et al., 2021). Our study calculated the tumor microenvironment score of osteosarcoma by the ESTIMATE algorithm, and analyzed the relationship between them and risk score or the degree of immune cell infiltration (Supplementary Figure 5). Differential analysis results showed that the immunoscore, stromal score, and Estimate score of the low-risk group were higher than those of the high-risk group (Figures 9A–C). Correlation analysis results showed that risk score was negatively correlated with immune score, stromal score, and Estimate score. With the increase of risk score, the immune score, stromal score, and Estimate score of osteosarcoma gradually decreased (Figures 9D–F). Conversely, the purity of tumor cells in the low-risk osteosarcoma group was lower than that in the high-risk group, and the purity of tumor cells was negatively correlated with the risk score (Figures 9G,H).

### Relationship Between Immune-Related LncRNAs Signature and Immune Escape in Osteosarcoma

**FIGURE 10:**
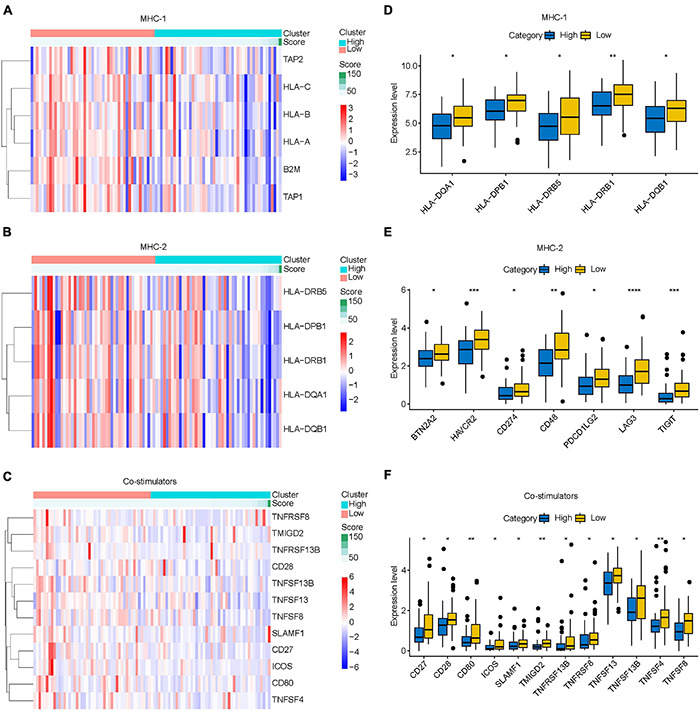
Relationship between IRLs signature and endogenous immune escape in osteosarcoma. **(A–C)** The expression of MHC-I molecule, MHC-II molecule, and costimulatory molecule changed with the increase of risk score. **(D–F)** Differential analysis of the expression of MHC-I, MHC-II, and costimulatory molecules in high-risk and low-risk osteosarcoma. *P < 0.05; **P < 0.01; ***P < 0.001; ****P < 0.0001.

Immune escape is an important reason for the rapid growth of tumor cells in the human body. In this study, we found that the prognosis of osteosarcoma may be related to the mechanism of endogenous immune escape. The molecules involved in endogenous immune escape include MHC-I molecules, MHC-II molecules, costimulatory molecules. Our results showed that the expression levels of MHC-I molecules, MHC-II molecules, and costimulatory molecules decreased with the increase of risk score (Figures 10A–C), and the different analysis results showed that the expression levels of these molecules in the high-risk group were lower than those in the low-risk group (Figures 10D–F). This suggests that immune escape is more likely to occur in the high-risk group osteosarcoma.

## Discussion

Compared with previous amputations alone, the survival rate for osteosarcoma has improved from 20% to more than 60% (Koster et al., 2018; Wang et al., 2018) but has not improved further in recent years. The high incidence of pulmonary metastasis is one of the important reasons. Therefore, searching for effective prognostic markers is an important method for early diagnosis of the prognosis of osteosarcoma and early intervention. In recent years, the role of TIM in the treatment of osteosarcoma has become increasingly evident. A growing number of immunotherapy agents are entering clinical trials (NCT04668300, NCT04544995, and NCT02500797). Therefore, we hope to find immune-related biomarkers that can not only better predict patient survival, but also provide potential therapeutic targets for future immunotherapy. LncRNA has been proved to be involved in the immune response of osteosarcoma by regulating the expression of TNF-α (Xu et al., 2020). To this end, we constructed a prognostic signature using IRLs to explore its role in the prognosis of osteosarcoma. Further, we explored the relationship between the IRLs signature and the TIM of osteosarcoma.

In this study, 10 IRLs with good predictive ability were selected as a prognostic marker for osteosarcoma by iterative LSSSO regression and multivariate Cox regression analysis. Compared with the simple univariate and multivariate Cox regression analysis, the IRLs signature has better predictive performance, with an AUC of 0.96. The results of PCA showed that IRLs signature could classify osteosarcoma into two groups. With the increase of risk score, the mortality of patients with osteosarcoma showed a significant upward trend. Recurrence and metastasis are important clinical features for the prognosis of osteosarcoma. Compared with the common clinical features of osteosarcoma, IRLs signature has higher predictive power for the prognosis of osteosarcoma, even higher than the two clinical features of osteosarcoma recurrence and metastasis.

Our study showed that the metastatic rate of osteosarcoma in the high-risk group was significantly higher than that in the low-risk group, and the probability of metastasis tended to increase with the increase of risk score. It is worth noting that the differential expression analysis of lncRNA in IRLs signature between the metastatic group and the non-metastatic group showed that only the expression level of AC006033.2 was different. Therefore, our study further verified the role of AC006033.2 in the biological behavior of osteosarcoma through an *in vitro* experiment. By interfering with AC006033.2, we found that the proliferation, migration, and invasion of osteosarcoma cell lines were enhanced. Studies have shown that AC006033.2 is also an effective biomarker for anti-tumor immunity in gastric cancer (He and Wang, 2020), which is similar to the results of our study. We found that IRLs signature is closely related to the TIM of osteosarcoma. In addition, the expression level of AC006033.2 was significantly positively correlated with the content of various immune cells. Therefore, AC006033.2 may be an important molecule affecting the prognosis of osteosarcoma.

LncRNA THRIL has been shown to regulate TNF-α expression and participate in immune response in osteosarcoma (Xu et al., 2020). KEGG and GO enrichment analysis results also showed that osteosarcoma in the low-risk group was highly correlated with immune pathways and functions. Therefore, we further explored the relationship between IRLs signature and TIM of osteosarcoma. Firstly, the ssGSEA was used in the study to evaluate the level of immune cell infiltration in the microenvironment of osteosarcoma, and the level of immune cell infiltration was higher in the low-risk group. Results of the ESTIMATE algorithm also indicated a higher immune score in osteosarcoma in the low-risk group. These suggest that IRLs may be associated with the immune cell infiltration in the microenvironment of osteosarcoma.

Studies have shown that lncRNA can also act on T cells to promote the immune escape of tumor cells (Huang et al., 2018). Therefore, we further explored the relationship between the IRLs signature and the TIM of osteosarcoma. The decrease of MHC-I molecular presentation function is considered to be an important reason for the immune escape of tumor cells (Lee et al., 2005). Low expression of MHC-II molecules can also lead to the occurrence of immune escape due to the inability to effectively activate T cells (Zhi et al., 2021). In addition, the activation of T cells also requires the participation of costimulatory molecules (Angulo et al., 2021). We found that MHC-I molecules, MHC-II molecules, and costimulatory molecules, which are associated with endogenous immune escape, were all low expressed in the high-risk group. These results suggest that immune escape may be prevalent in high-risk group osteosarcoma, and IRLs may be associated with the immune escape of osteosarcoma cells.

## Conclusion

The IRLs signature is a reliable biomarker for the prognosis of osteosarcoma, and they alter the prognosis of osteosarcoma. In addition, IRLs signature and patient prognosis may be related to TIM in osteosarcoma. The higher the content of immune cells in the TIM of osteosarcoma, the lower the risk score of patients and the better the prognosis. The higher the expression of immune escape-related genes, the lower the risk score of patients and the better the prognosis.

## Data Availability Statement

The datasets presented in this study can be found in online repositories. The names of the repositories and accession numbers can be found in the article material.

## Author Contributions

WG conceived the project. QH, YL, CC, JL, TR, YH, HZ, YY, YG, WW, BW, JN, JX, and LG performed the literature search and data analysis. QH and WG drafted and critically revised the work. All authors contributed to manuscript revision, read, and approved the submitted version.

## Conflict of Interest

The authors declare that the research was conducted in the absence of any commercial or financial relationships that could be construed as a potential conflict of interest.

## Publisher’s Note

All claims expressed in this article are solely those of the authors and do not necessarily represent those of their affiliated organizations, or those of the publisher, the editors and the reviewers. Any product that may be evaluated in this article, or claim that may be made by its manufacturer, is not guaranteed or endorsed by the publisher.

## Footnotes

1
https://ocg.cancer.gov/programs/target

2
http://www.gsea-msigdb.org/gsea/msigdb/index.jsp

## Abbreviations

EdU: 5-ethynyl-2 ′ -deoxyuridine
FBS: fetal bovine serum
GSEA: gene set enrichment analysis
GSVA: Gene Set Variation Analysis
IRLs: immune-related lncRNAs
PCA: principal component analysis
ssGSEA: single sample gene set enrichment analysis
TIM: tumor immune microenvironment.
