# Supplementary material for: Immune-Related LncRNAs Affect the Prognosis of Osteosarcoma, Which Are Related to the Tumor Immune Microenvironment
Source: Front Cell Dev Biol. 2021 Oct 7;9:731311. doi: 10.3389/fcell.2021.731311 (PMC8529014; doi:10.3389/fcell.2021.731311)
Supplement: Supplementary file 1 [file Table_1.DOCX]

| **Primers** | **Sequences** | |
| --- | --- | --- |
| AC006033.2 | Forward (5'-3') | CCTTCGCAGCCCACCAAATCC |
|  | Reverse (5'-3') | CTCCCACCCCACGCACAAAAG |
| GAPDH | Forward (5'-3') | GAAGGTCGGAGTCAACGGATTT |
|  | Reverse (5'-3') | ATGGGTGGAATCATATTGGAAC |

**Table S1** Primers for real-time PCR
