# Supplementary material for: Immune-Related LncRNAs Affect the Prognosis of Osteosarcoma, Which Are Related to the Tumor Immune Microenvironment
Source: Front Cell Dev Biol. 2021 Oct 7;9:731311. doi: 10.3389/fcell.2021.731311 (PMC8529014; doi:10.3389/fcell.2021.731311)
Supplement: Supplementary file 5 [file Table_5.DOCX]

**Table S5** Univariate Cox regression analysis was performed on 10 immune-related lncRNAs.

| id | HR | HR.95L | HR.95H | *p*-value |
| --- | --- | --- | --- | --- |
| AC006033.2 | 0.190087 | 0.062204 | 0.580881 | 0.003579 |
| AL133523.1 | 0.294434 | 0.111207 | 0.779551 | 0.013845 |
| SNHG6 | 1.688596 | 1.209821 | 2.356843 | 0.002073 |
| AC015795.1 | 3.473617 | 1.703031 | 7.085024 | 0.000617 |
| AP000943.1 | 2.671755 | 1.427753 | 4.999657 | 0.002114 |
| AC016746.1 | 2.000228 | 1.206954 | 3.314883 | 0.007150 |
| USP30-AS1 | 0.299942 | 0.121258 | 0.741933 | 0.009162 |
| LINC02315 | 0.505447 | 0.244326 | 1.045638 | 0.065821 |
| AC079760.2 | 0.481719 | 0.240863 | 0.963423 | 0.038892 |
| LINC01976 | 1.791782 | 1.156338 | 2.776422 | 0.009053 |
