# Supplementary material for: Immune-Related LncRNAs Affect the Prognosis of Osteosarcoma, Which Are Related to the Tumor Immune Microenvironment
Source: Front Cell Dev Biol. 2021 Oct 7;9:731311. doi: 10.3389/fcell.2021.731311 (PMC8529014; doi:10.3389/fcell.2021.731311)
Supplement: Supplementary file 6 [file Table_6.DOCX]

| id | coef | HR | HR.95L | HR.95H | *p*-value |
| --- | --- | --- | --- | --- | --- |
| AC006033.2 | -1.02671 | 0.358184 | 0.082315 | 1.558601 | 0.171169 |
| AL133523.1 | -1.04999 | 0.349941 | 0.132041 | 0.927425 | 0.034731 |
| SNHG6 | 0.460555 | 1.584953 | 0.984244 | 2.552289 | 0.058141 |
| AC015795.1 | 0.294893 | 1.342983 | 0.476435 | 3.785619 | 0.577032 |
| AP000943.1 | 1.275642 | 3.580999 | 0.728943 | 17.59198 | 0.116257 |
| AC016746.1 | 0.795515 | 2.215583 | 0.816015 | 6.015582 | 0.118525 |
| USP30-AS1 | -0.70552 | 0.493852 | 0.174178 | 1.400232 | 0.184556 |
| LINC02315 | -1.53329 | 0.215825 | 0.090988 | 0.511939 | 0.000503 |
| AC079760.2 | -1.32196 | 0.266612 | 0.100217 | 0.709278 | 0.008096 |
| LINC01976 | 0.928334 | 2.530289 | 1.351382 | 4.737641 | 0.003720 |

**Table S6** Multivariate Cox regression analysis was performed on 10 immune-related lncRNAs.
