# Supplementary material for: Immune-Related LncRNAs Affect the Prognosis of Osteosarcoma, Which Are Related to the Tumor Immune Microenvironment
Source: Front Cell Dev Biol. 2021 Oct 7;9:731311. doi: 10.3389/fcell.2021.731311 (PMC8529014; doi:10.3389/fcell.2021.731311)
Supplement: Supplementary file 7 [file Table_7.DOCX]

| Clinical features | HR | HR.95L | HR.95H | *p*-value |
| --- | --- | --- | --- | --- |
| Gender | 0.971681 | 0.506002 | 1.865931 | 0.931234 |
| Age | 0.980766 | 0.919763 | 1.045814 | 0.553335 |
| Metastasis | 3.805849 | 1.985854 | 7.293833 | 5.65E-05 |
| Relapse | 17.30436 | 5.297624 | 56.52361 | 2.35E-06 |
| Site | 0.104204 | 0.037558 | 0.289115 | 1.40E-05 |
| Risk score | 1.024151 | 1.014063 | 1.034339 | 2.30E-06 |

**Table S7** Univariate Cox regression analysis was performed on the risk score and clinical features of osteosarcoma.
