# Supplementary material for: Immune-Related LncRNAs Affect the Prognosis of Osteosarcoma, Which Are Related to the Tumor Immune Microenvironment
Source: Front Cell Dev Biol. 2021 Oct 7;9:731311. doi: 10.3389/fcell.2021.731311 (PMC8529014; doi:10.3389/fcell.2021.731311)
Supplement: Supplementary file 8 [file Table_8.DOCX]

| id | HR | HR.95L | HR.95H | *p*-value |
| --- | --- | --- | --- | --- |
| Gender | 0.560661 | 0.262066 | 1.19947 | 0.135900 |
| Age | 1.110481 | 1.039228 | 1.186619 | 0.001953 |
| Metastasis | 3.394051 | 1.617057 | 7.123792 | 0.001236 |
| Relapse | 23.63708 | 6.273096 | 89.06474 | 2.97E-06 |
| Site | 0.120369 | 0.039946 | 0.362706 | 0.000169 |
| Risk score | 1.020501 | 1.008734 | 1.032406 | 0.000605 |

**Table S8** Multivariate Cox regression analysis was performed on the risk score and clinical features of osteosarcoma.
