# Supplementary material for: Immune-Related LncRNAs Affect the Prognosis of Osteosarcoma, Which Are Related to the Tumor Immune Microenvironment
Source: Front Cell Dev Biol. 2021 Oct 7;9:731311. doi: 10.3389/fcell.2021.731311 (PMC8529014; doi:10.3389/fcell.2021.731311)
Supplement: Supplementary file 11 [file Data_Sheet_1.PDF]

Supplementary Figures

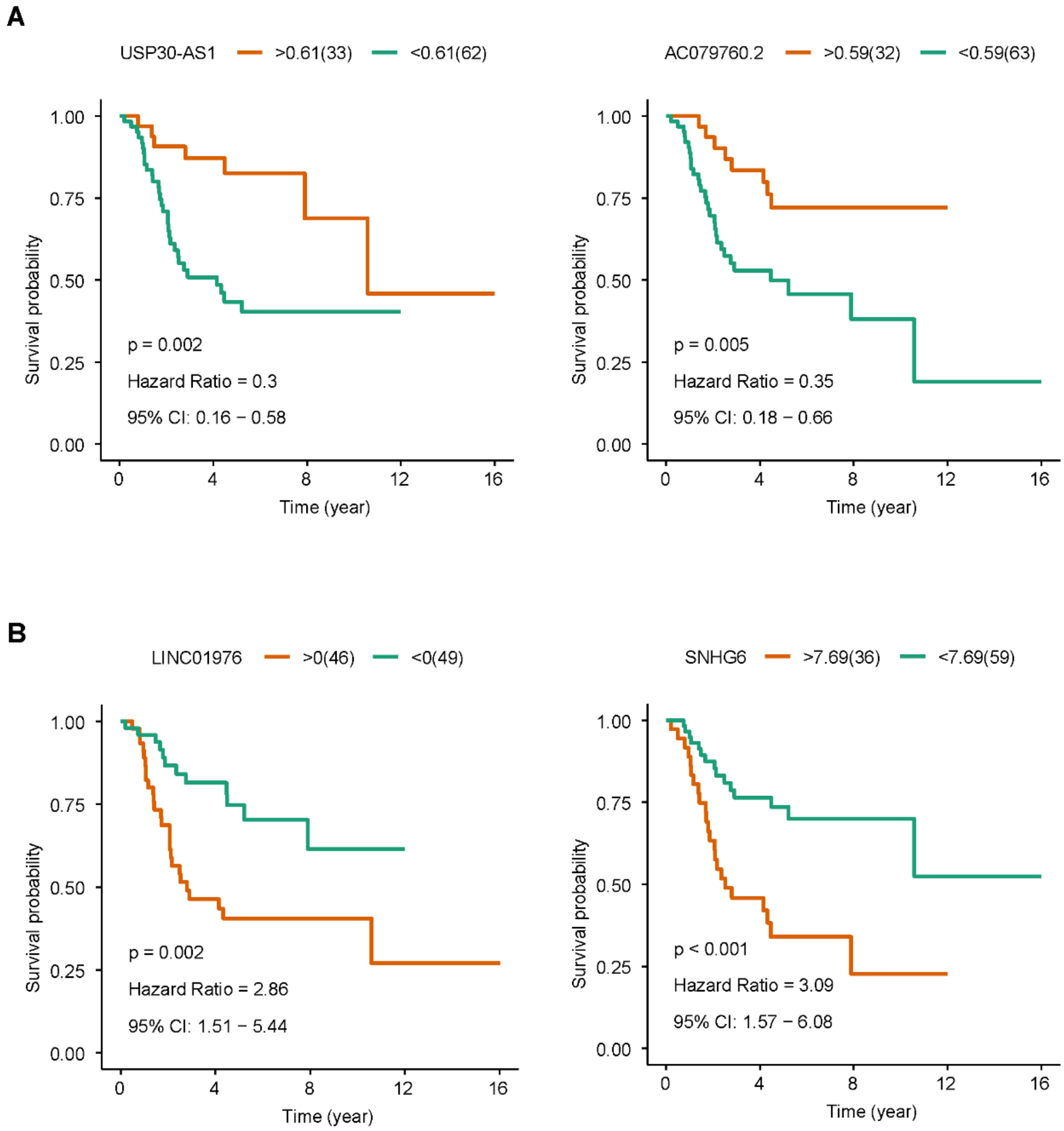

Figure S1 Role of IRLs signature in the prognosis of osteosarcoma. A, B Osteosarcomas were grouped according to the optimal cut-off value of lncRNAs expression in IRLs signature, and survival analysis were performed.

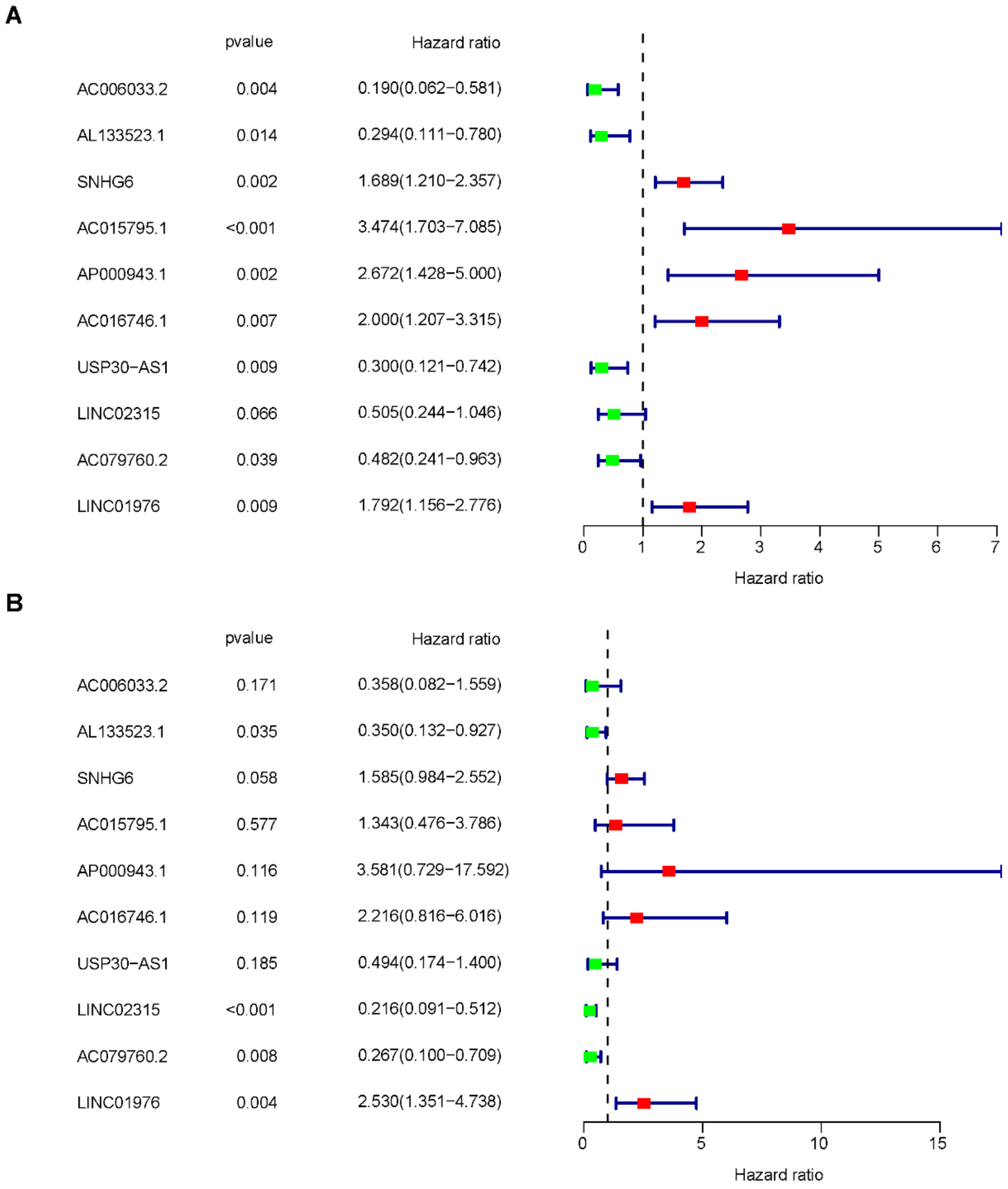

Figure S2 Independent prognostic analysis of 10 IRLs in IRLs signature of osteosarcoma. A. Univariate Cox regression analysis of 10 IRLs. **B.** Multivariate Cox regression analysis of 10 IRLs.

A

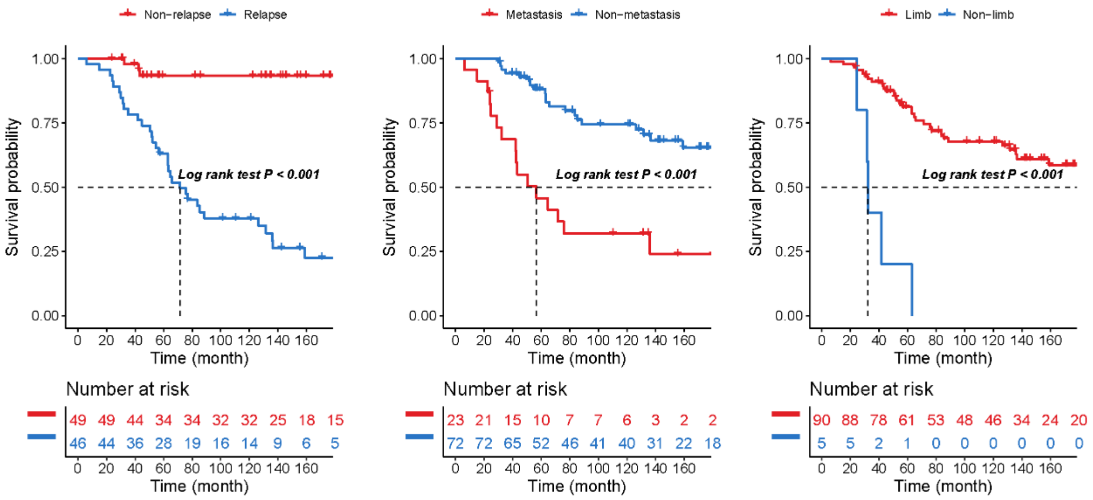

B

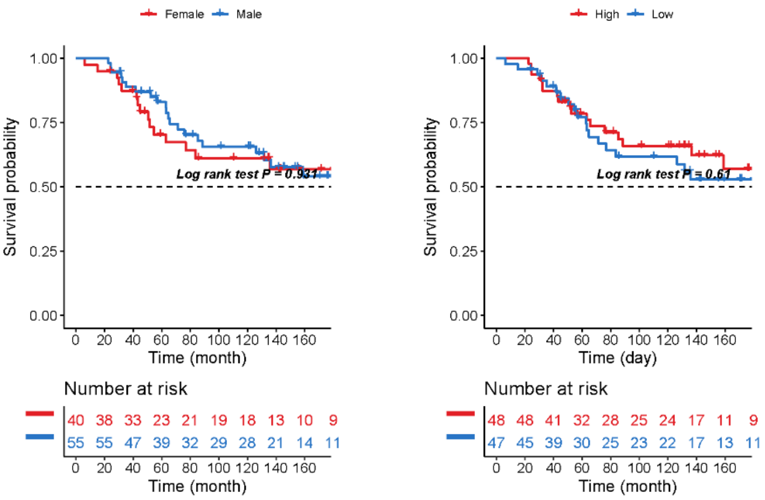

Figure S3 Role of clinical features in the prognosis of osteosarcoma. A. Influence of recurrence, metastasis and tumor location on the prognosis of osteosarcoma. **B.** Influence of gender and age of patients with osteosarcoma on the prognosis of osteosarcoma.

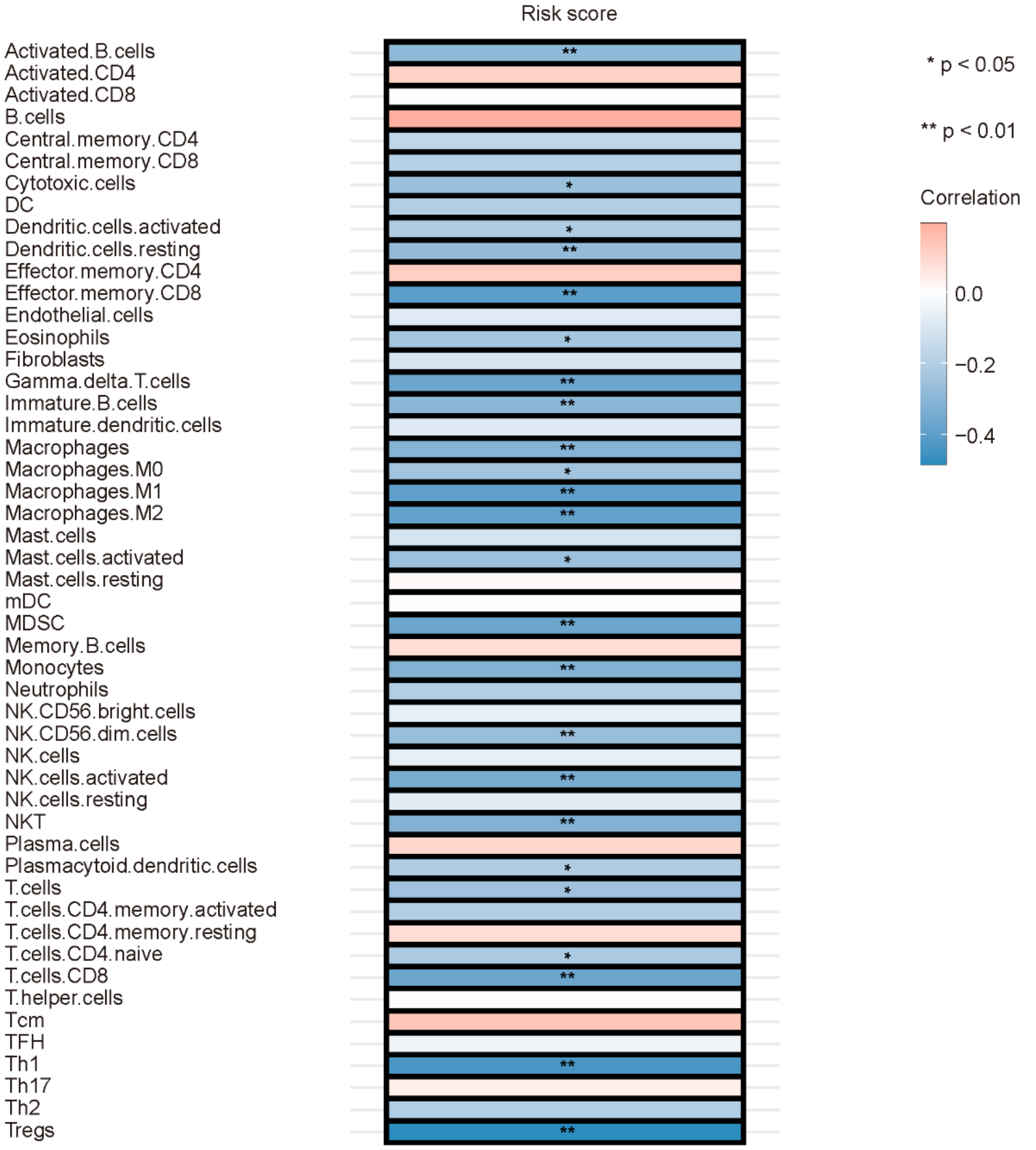

Figure S4 Correlation analysis between risk score and the degree of immune cell infiltration in osteosarcoma.

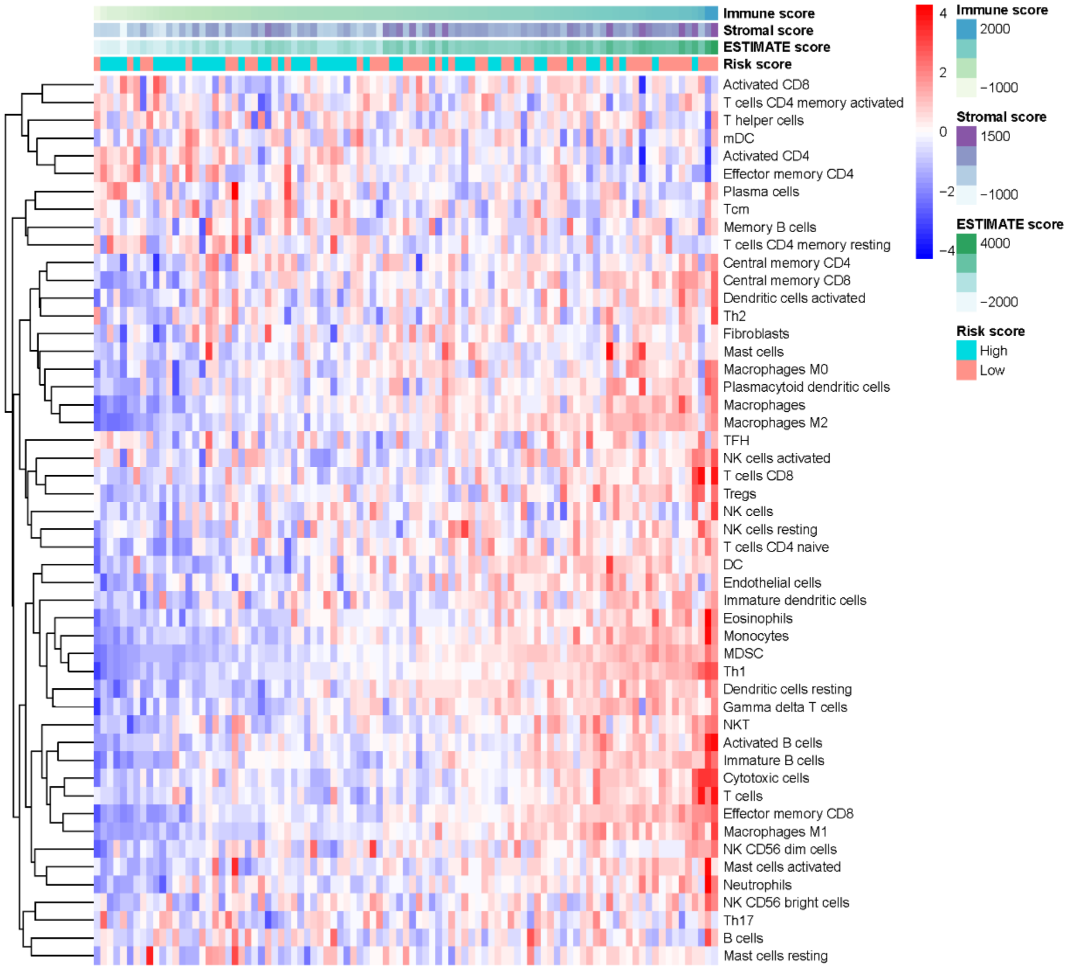

Figure S5 Relationship between tumor microenvironment score, risk score, and immune cell infiltration degree in Osteosarcoma.
